# Interaction of MG63 Human Osteosarcoma-Derived Cells on S53P4 Bioactive Glass: An In Vitro Study

**DOI:** 10.3390/jfb16080275

**Published:** 2025-07-29

**Authors:** Valentin Schmidt, Beáta Polgár, Vanda Ágnes Nemes, Tímea Dergez, László Janovák, Péter Maróti, Szilárd Rendeki, Kinga Turzó, Balázs Patczai

**Affiliations:** 1 Department of Traumatology and Hand Surgery, Faculty of Medicine, University of Pécs, Ifjúság Str. 13, H-7624 Pécs, Hungary; vanda.nemes@aok.pte.hu (V.Á.N.); patczai.balazs@pte.hu (B.P.); 2 Department of Medical Microbiology and Immunology, Faculty of Medicine, University of Pécs, Szigeti Str. 12, H-7624 Pécs, Hungary; polgar.beata@pte.hu; 3 Insitute of Bioanalysis, Faculty of Medicine, University of Pécs, Honvéd Str. 1, H-7624 Pécs, Hungary; timea.dergez@aok.pte.hu; 4 Department of Physical Chemistry and Materials Science, University of Szeged, Rerrich Béla Square 1, H-6721 Szeged, Hungary; laszlo.janovak@gmail.com; 5 3D Printing and Visualisation Centre, University of Pécs, Boszorkány Str. 2, H-7624 Pécs, Hungary; peter.maroti@aok.pte.hu; 6 Medical Simulation Education Centre, Faculty of Medicine, University of Pécs, Szigeti Str. 12, H-7624 Pecs, Hungary; szilard.rendeki@aok.pte.hu; 7 Dental School, Faculty of Medicine, University of Pécs, Tüzér u.1, H-7623 Pécs, Hungary; turzo.kinga@pte.hu

**Keywords:** bioactive glass, BonAlive^®^ granules, hip and knee prosthesis, medical implants, MG63 osteoblast-like cells

## Abstract

Bioactive glass materials have been used for decades in orthopedic surgery, traumatology, and oral and maxillofacial surgery to repair bone defects. This study aimed to evaluate in vitro the survival and proliferation of MG63 human osteosarcoma-derived cells on S53P4 bioactive glass (BonAlive^®^ granules). Microscopic visualization was performed to directly observe the interactions between the cells and the material. Osteoblast-like cells were examined on non-adherent test plates, on tissue culture (TC)-treated plates and on the surface of the bioglass to assess the differences. Cell survival and proliferation were monitored using a CCK-8 optical density assay. Comparing the mean OD of MG63 cells in MEM on TC-treated plates with cells on BG, we detected a significant difference (*p* < 0.05), over each time of observation. The sustained cell proliferation confirmed the non-cytotoxic property of the bioglass, as the cell number increased continuously at 48, 72, 96, and 168 h and even did not plateau after 168 h. Since the properties of bioglasses can vary significantly depending on their composition and environment, a thorough characterization of their biocompatibility is crucial to ensure their effective and appropriate application—for example, during hip and knee prosthesis insertion.

## 1. Introduction

Large bone defects represent significant discontinuities in bone tissue structure and therefore make spontaneous healing impossible and present substantial clinical challenges [1]. These defects are commonly caused by traumatic injuries, severe bone infections, congenital diseases, tumor resections, or other surgical interventions [1,2,3].

Bioactive glasses (BGs) are considered suitable biomaterials for bone reconstruction due to their osteoconductive and osteoinductive properties. They are mainly used in spinal fusion surgeries, oral and maxillofacial procedures, and the treatment of long bone fractures as bone substitute in cases of bone defects [4].

By definition, a bioactive material is one that triggers a specific biological response at its interface, leading to the formation of a bond between the material and the host tissue [5]. A common feature of bioactive materials is their ability to form a carbonated hydroxyapatite layer on their surface, which is chemically and structurally similar to the mineral of natural bone. This is a crucial step for osseo- and biointegration [6]. Furthermore, numerous comparative studies have demonstrated the superior ability of bioglasses to stimulate cellular responses compared to other Ca-P ceramics, such as hydroxyapatite (HA) [7].

**Figure 1:**
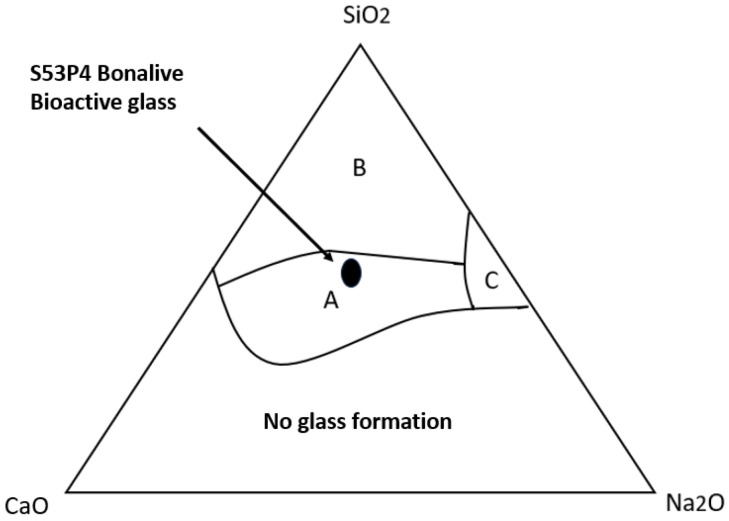
Grouping of different glass materials according to their composition in a triangular system. The regions correspond to different types of glasses: bioactive glass (A), inert glass (B), or water glass (C). S53P4 BonAlive bioactive glass is highlighted in the bioactive region with a black oval. A: BGs are chemically bound to the bone (approx. 6 w% P_2_O_3_ content). B: a fibrous capsule forms on the surface of inert glasses (e.g., window glass) in a tissue environment. C: biodegradable glasses are absorbed and replaced by the surrounding tissues [9].

The fundamental biological properties and behavior of BG, such as absorption and ion release, are primarily determined by its composition. The different glass-ceramics can be categorized and represented using a triangular classification system, as shown in Figure 1. Silicate BGs (e.g., 45S5 Bioglass) exhibit strong bioactivity. They promote the release of soluble Si, Ca, P, and Na ions at their surface, triggering cellular responses that induce bone formation and enhance the biointegration of orthopedic prostheses. Soluble silicone enhances calcification and stimulates osteoblast activity. Phosphate- and borate-based BGs dissolve and absorb easily [8].

The basic biological properties and behavior of bioglass, such as absorption and ion release, are determined by its composition and environment. The ion-releasing effect of BG induces osteogenic and angiogenic responses. Silicate BGs (e.g., 45S5 Bioglass) promote the release and exchange of soluble Si, Ca, P, and Na ions on the surface of the material. This leads to specific cellular responses inducing bone formation, making them favorable for the biointegration of orthopedic prostheses. Phosphate- and borate-based BGs exhibit a pronounced ability to dissolve and absorb, whereas silicate-based BGs display strong “bioactive” behavior. Soluble silicone enhances calcification and stimulates osteoblast activity [9].

Due to their suboptimal mechanical properties such as rigidity, low bending strength, and limited fracture resistance, the usage of BGs in load-bearing applications is restricted [4,10]. The weaknesses also include reduced vascularization and an increased risk of inflammation [11,12,13]. Although BGs present intrinsic antibacterial properties [14], there is a need to strengthen these features, with the goal of reducing bacterial infections [15].

Nevertheless, the demand for BGs has grown, especially with the growing number of hip, knee, and dental implant placements, and to date, more than 1.5 million patients worldwide have been treated with Bioglass^®^ 45S5 [16].

BGs are also used as coatings for metal implants, combining the mechanical strength of metals with the biocompatibility and bioactivity of glasses [17]. The modern definition of biocompatibility refers to the acceptance of an artificial implant by the surrounding tissues and the whole body [9].

For implantable BGs, adherence to regulatory frameworks is crucial, particularly those established by the Food and Drug Administration (FDA) in the United States and the Medical Device Regulation (MDR) within the European Union (EU). Under MDR, BG implants are classified as Class III medical devices due to their high-risk nature and direct contact with internal tissues [18]. International ISO standards are also important as they provide comprehensive guidance on material properties, biocompatibility requirements, and manufacturing processes, ensuring the safety and quality of implantable materials [18,19].

The ISO 10993 family of standards provides a comprehensive framework for evaluation to address biological risks, aiming to prevent harmful reactions and meet the safety requirements for medical devices [20]. This guidance emphasizes the importance of evaluating biocompatibility of medical devices in their final, sterilized form, where applicable [18,21]. One of the primary (initial) biocompatibility assessments is cytotoxicity testing, which is essential in the development of any biomaterial, with the advantage that fewer animal experiments are required before clinical trials [22].

MG63 cells are widely used in osteogenesis research due to their stable phenotype across multiple passages, making them a suitable in vitro model for assessing the cytotoxicity and biocompatibility of implant materials [23].

The aim of the present work was to evaluate in vitro the survival and proliferation of MG63 human osteosarcoma-derived cells on S53P4 (BonAlive^®^) BG granules, using the CCK-8 optical density (OD) assay.

Yoo et al. (2024) have described the bioresorption and biomineralization of S53P4 BG in neutral Tris buffer and citric acid solution [24]. MG63 cells’ viability was assessed after 24 h using a CCK-8 cell-counting kit. Our study adds important information regarding the proliferation of MG63 osteoblast-like cells after 48 h, 72 h, 96 h, and 168 h. Additionally, we performed inverted microscopic images of these osteoblast-like cells on various surfaces: non-treated plates, TC-treated plates, and BG. Microscopic images revealed spheroid formation, detailing the surface-dependent behavior and survival patterns of these cells.

## 2. Materials and Methods

### 2.1. Sample Preparation

**Figure 2:**
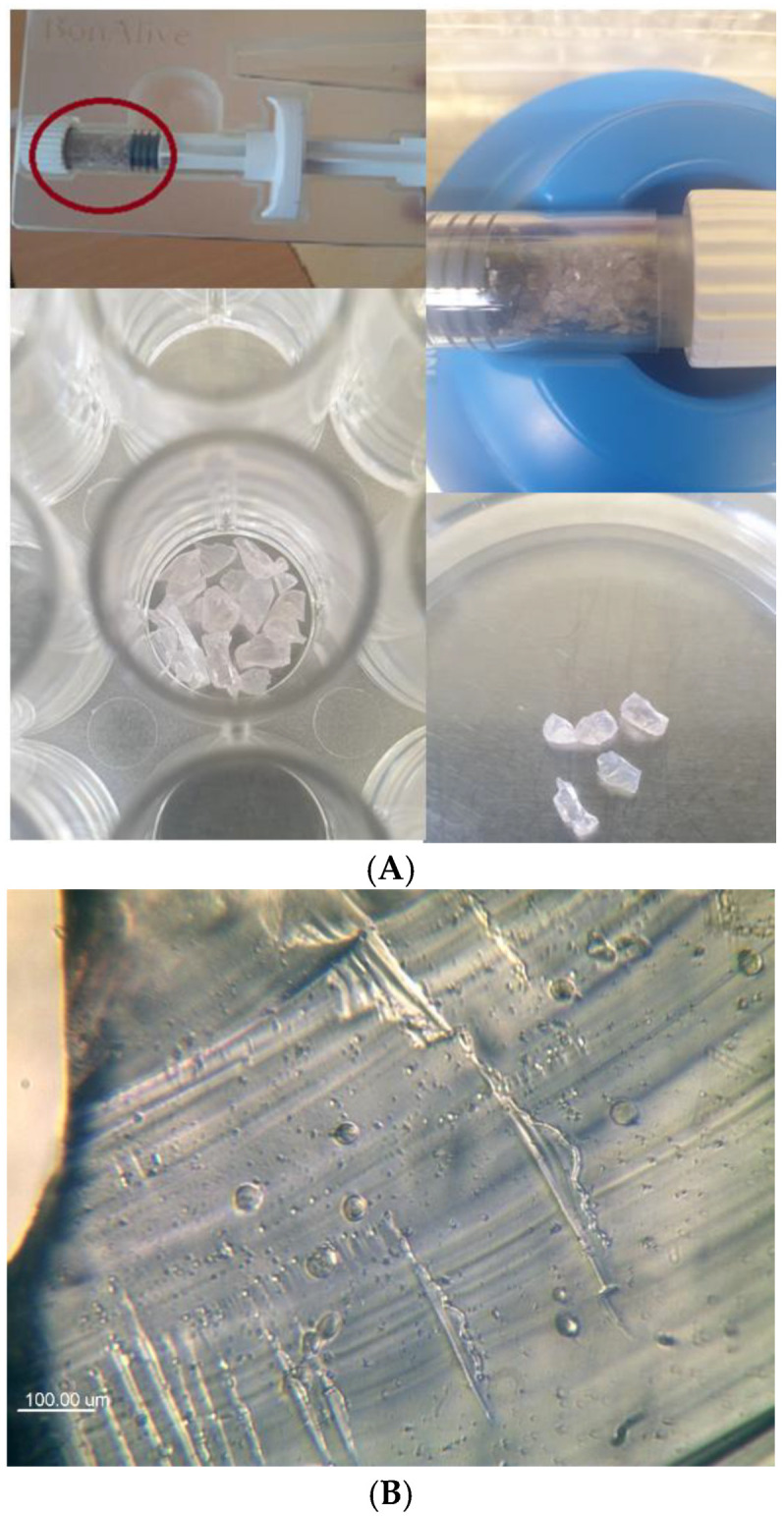
(**A**) BonAlive^®^ S53P4 granules in their original holder and in cell culture plates (BonAlive Biomaterials Ltd. Finland). (**B**) Inverted microscopic image of BonAlive surface (×10 objective), with spherical MG63 osteoblast-like cells on the surface at 24 h observation.

All procedures were carried out in a laminar flow cabinet (Forma Scientific Inc., Class IIA/B3 Biological Safety, Marietta, OH, USA) under sterile conditions, using sterile tools to ensure a contaminant-free environment for tissue culture analysis and testing. A sterile spatula was used to place the bioglass granules (BonAlive^®^ S53P4 granules, 1–2 mm, sterile; BonAlive Biomaterials Ltd., Turku, Finland; Figure 2) into the test holes of a 48-well sterile culture plate, ensuring complete coverage of the well bottoms. The study was performed on two different culture plates: a standard, flat-bottom, tissue culture (TC)-treated plate (Type: Falcon 353078, Corning-Materials Science Technology and Innovation, Charlotte, NC, USA) for positive control tests, allowing cell adhesion to the bottom of the wells, and a non-treated, non-adherent plate (Type: VWR 734-2780, Avantor, Inc., Radnor, PA, USA) for definitive BG examinations, where cells did not adhere to the well of the plate but only to the BG granules.

### 2.2. Preconditioning of BonAlive Granules

BGs rapidly exchange ions with their surrounding environment, leading to a rise in pH under static in vitro conditions due to the “burst release” of alkaline ions, as described earlier [6]. This phenomenon presents difficulties in cell culture studies, due to its cytotoxic effects. A common method to lessen this issue is preconditioning, which involves immersing BG samples in an aqueous solution for a specific period [25]. In our study, Eagle’s Minimum Essential Medium (MEM) (Merck Group, Sigma-Aldrich GmbH, Darmstadt, Germany) was used for preconditioning.

The process was carried out as follows. First, sterile bioglass granules were placed into the holes of a 48-well tissue culture plate. Each well was then filled with 500 μL/well of complete Eagle’s MEM medium, with 1% non-essential amino acids (Merck Group, Sigma Aldrich GmbH, Darmstadt, Germany), 2 mm UltraGlutamine (Lonza Group AG, Basel, Switzerland), 1 mm sodium-pyruvate (Gibco, Thermo Fisher Scientific Inc., Waltham, MA, USA), 10% Fetal Bovine Serum (FBS, qualified, Gibco, Thermo Fisher Scientific Inc., Waltham, MA, USA), and 1% Penicillin–Streptomycin solution (P/S) (Lonza Group AG, Basel, Switzerland). The glass-coated plates were incubated in a humidified environment for 60 h at 37 °C with 5% CO_2_. After the incubation period, the nutrient medium was completely removed, and the preconditioned granules were immediately used for cell culture experiments.

### 2.3. Cell Culture

MG63 osteosarcoma-derived cell lines were obtained from the European Collection of Cell Cultures (UK). The frozen ampule was transferred to a 37 °C water bath for 1–2 min. The contents of the ampule were gently centrifuged in complete MEM medium to remove the cryoprotectant agent (DMSO). The harvested cells were then pipetted into a 25 cm^2^ flask containing 10 mL complete MEM medium. The MG63 cell cultures reached confluence within 2–4 days. Confluent cultures were rinsed twice with PBS and harvested by a 2–4 min long trypsinization using 0.25% trypsin–EDTA solution (Lonza Group AG, Basel, Switzerland). The obtained cells were then resuspended in complete MEM nutrient medium and maintained under standard conditions at 37 °C under a humidified atmosphere containing 5% CO_2_.

### 2.4. Microscopic Images of BG and MG63 Osteoblast-Like Cells

Microscopic images were captured after 24 h, 48 h, 72 h, 96 h, and 168 h from the surface of BonAlive granules and MG63 osteoblast-like cells with a Leica DMIL inverted microscope (type 090-131.001; Leica Mikroskopies & Systeme GmbH, Wetzlar, Germany), a ×10 objective, and a VisiCam 3.0 USB camera (Cat: 630-1031) with 3 Mpixel and a resolution of 2048 × 1536 pixels (VWR International BVBA, Leuven, Belgium). Image analyzer program: VisiCam Image Analyzer v6236 March 2010. Cells were observed on different substrates, described in the following chapter.

### 2.5. CCK-8 Assay to Establish the Proliferation Rate of the Cells

MG63 cells near confluence were harvested and seeded in 500 μL/well complete MEM medium into 48-well TC-treated or non-treated test plates at a density of 10,000 cells/well. For bioglass experiments, osteosarcoma-derived cells were seeded directly onto the preconditioned bioglass surface. The optimal cell density was determined in preliminary calibration experiments. Cells at passage numbers 8–15 were used for all assays.

The following test conditions were used during the cell culture experiments:

I. Non-treated (non-adherent) test plates: (1) complete MEM medium alone without cells (to assess background CCK-8 reactivity); (2) complete MEM medium with bioglass particles; (3) MG63 cells seeded onto bioglass granules in complete MEM medium; (4) MG63 cells placed into wells without bioglass granules.
II. TC-treated test plates: (1) complete MEM medium alone without cells (for background CCK-8 reactivity); (2) MG63 cells in complete MEM medium (positive control) to verify cell viability and proliferation.

After seeding, the culture plates were incubated in a humidified atmosphere at 37 °C with 5% CO_2_. Cell adhesion was assessed after 24 h, and the proliferation was investigated 48 h, 72 h, 96 h, and 168 h following seeding. Four independent experiments were performed, with four samples for each treatment and assay.

The growth and viability of cultured MG63 cells was measured using the CCK-8 assay (Cell Counting Kit-8, Merck Group, Sigma-Aldrich GmbH, Darmstadt, Germany), a sensitive colorimetric method. At each time point (24, 48, 72, 96, and 168 h), the culture supernatant was carefully removed to avoid damaging the cells attached to the surface of the bioglass. It was then replaced with 200 μL/well of fresh, pre-warmed complete MEM medium and 20 μL/well of CCK-8 reagent. The CCK-8 reagent, originally pink in color, turns orange in the presence of proliferating cells. The intensity of the orange color, measured at 450 nm, is proportional to the number of living cells [26]. Following a 2 h incubation period at 37 °C in 5% CO_2_ in a humidified incubator, 100 μL of cell-free, CCK-8 containing supernatant was aspirated from the test wells and transferred to a 96-well microplate. The OD of the microplate wells was measured at 450/650 nm with a SpectoStar Nano Microplate Reader (BMG Labtech, Ortenberg, Germany).

### 2.6. Statistical Analyses

For the CCK-8 assay, the arithmetic means of OD measured at 450 nm ± the standard error of the mean (SE) was calculated. After normality testing, the data were compared via the Kruskal–Wallis test with the SPSS 29 program (IBM, New York, NY, USA). The probability value of *p* < 0.05 was considered statistically significant.

## 3. Results

### 3.1. Microscopic Characterization of BG and MG63 Osteoblast-Like Cells

This study served as a visual evaluation of the changes in BG granules and MG63 osteoblast-like cells on different substrates and at different timings. In parallel with the primary cytotoxicity test, a microscopic evaluation was performed. The preconditioned BonAlive granules (after 60 h of preconditioning) and the human osteosarcoma-derived MG63 cells were directly visualized after 24, 48, 72, 96, and 168 h of cultivation with a Leica DMIL inverted microscope.

**Figure 3:**
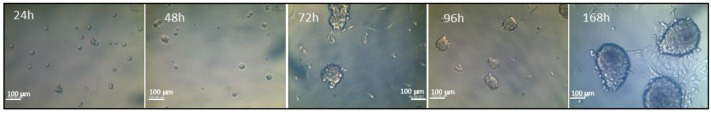
Inverted microscope images of MG63 cells grown on non-treated plates after 24, 48, 72, 96, and 168 h. Scale bar: 100 μm.

Figure 3 presents microscopic images of MG63 osteoblast-like cells on non-treated plates after 24 h, 48 h, 72 h, 96 h, and 168 h of observation time. After 24 h, we can observe typical spherical morphology of osteoblast cells. Increasing the time, spheroids start to form, and bigger aggregations of the cells can be seen.

**Figure 4:**
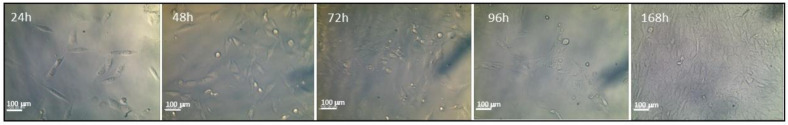
Inverted microscope images of MG63 cells on TC-treated plates after 24, 48, 72, 96, and 168 h. Scale bar: 100 μm.

Figure 4 presents microscopic images of MG63 osteoblast-like cells on TC-treated plates after 24 h, 48 h, 72 h, 96 h, and 168 h of observation time. Already after 24 h, we can observe that the morphology of osteoblast-like cells is not a spherical one, like on non-treated plates. The elongated form of the cells shows a better viability on the TC-treated surface. With increasing the time of observation, a confluent layer of cells formed. Spheroid formation was not detected on this surface.

**Figure 5:**
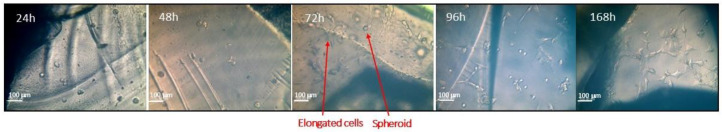
Inverted microscope images of MG63 cells on BonAlive granules (on non-treated plates) after 24, 48, 72, 96, and 168 h. Scale bar: 100 μm.

Figure 5 presents microscopic images of MG63 osteoblast-like cells on BonAlive granules on non-treated plates after 24 h, 48 h, 72 h, 96 h, and 168 h of observation time. After 72 h, small spheroids as well as elongated osteoblast-like cells can be seen. With increasing observation time, more elongated cells can be observed.

**Figure 6:**
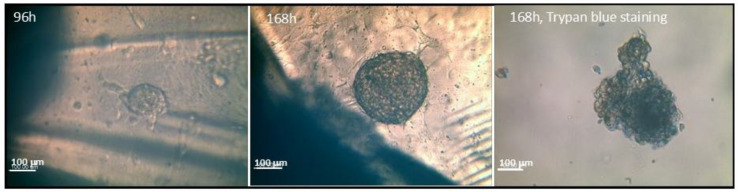
Inverted microscope images of MG63 cell spheroids on BonAlive granules (on non-treated plates) after 96 h and 168 h. A spheroid was stained with trypan blue. The dark blue color shows nonliving cells. Scale bar: 100 μm.

Figure 6 presents microscopic images of spheroids formed by MG63 osteoblast-like cells on BonAlive granules (on non-treated plates) after 96 h and 168 h of observation time. Spheroids formed on the surface of the glasses which grew with time. After 168 h, a spheroid was stained with trypan blue. This image shows several nonliving cells with dark blue color.

### 3.2. Investigation of the Proliferation of MG63 Cells on Bioglass Surface with CCK-8 Assay

A primary (initial) biocompatibility test was performed on BonAlive granules with an in vitro cell culture study of the human osteosarcoma-derived MG63 cell line.

**Figure 7:**
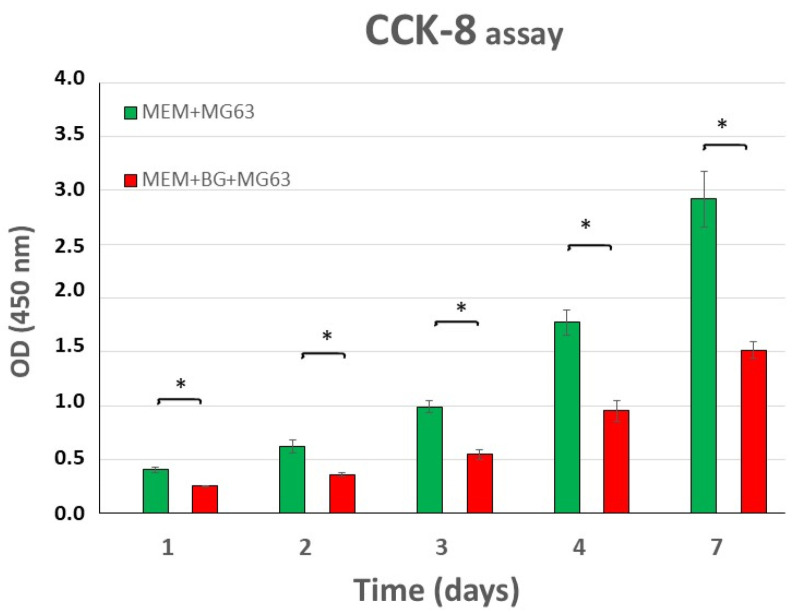
Bar graph illustrating the proliferation analysis of MG63 cells measured at different time points (24, 48, 72, 96, and 168 h) via the CCK-8 assay (OD values measured at 450 nm). Notations: MEM + MG63: OD of cells grown in medium on TC-treated plate; MEM + BG + MG63: OD of cells and BG on non-treated plate. Data are presented as mean ± the standard error of the mean (SE); asterisks denote significant differences (* *p* < 0.05).

Figure 7 and Table 1 gather the results of CCK-8 assay. The optical density (OD) of MG63 cells increased proportionally with the number of living cells over time. Bioglass OD was not different from the medium OD measured on the non-treated plate. MG63 osteoblast-like cells proliferated at a higher rate on the TC-treated control plate (MEM + MG63 on Figure 7) than on the bioglass surface (MEM + BG + MG63 on Figure 7).

The significant increase in the rate of proliferation demonstrates the viability of the investigated osteoblast-like cells on the bioglass surface (Figure 7, Table 1 and Table 2).

When comparing the mean OD of MG63 cells in MEM on TC-treated plates with cells on BG, we can see a significant difference (* *p* < 0.05), over each time of observation (Table 2). The same level of significance was detected after 24 h between the medium and the cells on BG (* *p* < 0.05). After 48, 72, 96, and 168 h, the cells were significantly proliferating on BG compared to the medium alone (** *p* < 0.01). A high significance (*** *p* < 0.001) was observed between the mean OD of MG63 cells and the mean OD of the medium, over each time of observation. A similar high significance (*** *p* < 0.001) was detected between the mean OD of MG63 cells and BG.

Table 2 shows the results (*p* values) of the pairwise comparisons of groups by the Kruskal–Wallis test performed with the SPSS 29 program (IBM, New York, NY, USA). The probability value of *p* < 0.05 was considered statistically significant. Asterisks denote the following significant differences: * *p* < 0.05, ** *p* < 0.01, and *** *p* < 0.001; N.S.: non-significant.

The most important finding is that when comparing the OD values of MEM + BG with the OD values of MEM + BG + MG63, a significant difference can be observed (* *p* < 0.05), all over each time of observations, proving that MG63 osteoblast-like cells survive and proliferate on BG granules.

## 4. Discussion

In this study, we examined the behavior of MG63 cells on the surface of BonAlive^®^ bioglass in an in vitro cell culture model. Two assessment methods were applied: (1) inverted microscope images were captured to visualize the changes in BG granules and MG63 osteoblast-like cells on different substrates and at different timings; (2) proliferation was assessed in four independent series, and the growth rates of osteosarcoma-derived cells on bioglass were determined by measuring optical density (OD) using the CCK-8 assay.

Our previous in vitro experiments showed that after “moistening” with sterile NaCl (0.9%), the BG formed a slightly moist mass. Afterwards, different antibacterial particles (Ag, Au, ZnO) were injected into the structure with NaCl (0.9%). None of these particles precipitated, and the sample remained homogeneous [27,28,29]. This is presumably due to the alkaline pH of the wetted BG and the high ionic strength resulting from the released Na, Ca, Si, and P ions [14]. These phenomena alone endow bioglass with antibacterial properties; however, this is not permanent, and the effect decreases over time. Incorporating antibacterial particles into the BG pore structure may help prevent infection recurrence in both the short and long term. Furthermore, our experiments with MG63 cells indicated that the crystals did not swell after preconditioning. When MG63 cells were seeded onto the BonAlive granules, they remained clearly visible, and the osteoblast-like cells were able to adhere and proliferate on their surface (Figure 5).

Our investigation shows that microscopic images supported the results of the CCK-8 assay. MG63 osteoblast-like cells can adhere to and proliferate on BonAlive^®^ bioglass. Comparing the two proliferation curves (MEM + MG63 vs. MEM + MG63 + BG), it can be stated that the cells on bioglass exhibit lower OD values (Figure 7 and Table 1), but almost half of the cells on the bioglass were capable of proliferation. This can be attributed to the fact that only the cells that come into direct contact with the bioglass survive and divide, while those that float to the bottom of the plate and cannot adhere will eventually die. Overall, bioglass provided a supportive surface for MG63 cell growth. Although osteoblast-like cells proliferate a little slower on bioglass compared to the plate, the rate remains satisfactory.

Inverted microscopic images revealed formation of spheroids of MG63 cells mainly on non-treated plates. On these plates, cells are unable to adhere permanently, so over time they round up, then form spheroids, and finally die (Figure 3). On TC-treated plates, the cells proliferated at the highest rate and reached their elongated form. This experimental condition provides a favorable environment for various cellular processes such as cell survival, spreading, and proliferation (Figure 4). After 168 h, a confluent layer of cells formed. Spheroid formation was not observed on the TC-treated plate. Microscopic images of MG63 osteoblast-like cells on BonAlive granules on non-treated plates revealed that, after 72 h, small spheroids formed beside elongated osteoblast-like cells (Figure 5). With longer observation times, more living cells could be seen, which supports the finding of the CCK-8 assay.

When observing spheroid formation on BonAlive granules after 96 h and 168 h, we could detect the growing of the spheroid, due to the aggregation of more cells (Figure 6). The staining with trypan blue of a spheroid after 168 h revealed that some cells were already not living (cells with dark blue color).

Spheroids were also visible on the non-treated tissue culture plate, meaning that their formation occurs even without the presence of bioglass. Therefore, the bioglass does not help their formation; they simply form due to the lack of a suitable surface for adhesion. Furthermore, the lifespan of the cells forming the spheroids is no longer in the presence of bioglass, as they die after 168 h.

Price et al., in 1998, examined the viability and proliferation of a human osteoblast cell line on Bioglass^®^ discs and titanium (Ti-6Al-4V) and cobalt–chrome–molybdenum (Co-Cr-Mo) alloys. They found significantly fewer cells on Co-Cr-Mo compared to Bioglass^®^, titanium, and polystyrene control surfaces (*p* < 0.05). Cell viability was also assessed by trypan blue exclusion using a hemocytometer, and detectable differences in cell morphology were observed on these biomaterials. Functional capacity was tested by measuring osteocalcin production using ELISA, and no significant differences were detected among the different biomaterials [30].

A similar in vitro study was performed by Yoo et al., (2024) in that respect, that MG63 cell viability was observed after 24 h using the CCK-8 cell-counting kit [24]. Besides that, they have characterized the bioresorption and biomineralization of S53P4 BG in neutral Tris buffer and citric acid solution. The morphology and particle size of S53P4 BG powder were determined by field emission scanning electron microscopy (FE-SEM), and compressive strength was tested with a Zwick mechanical testing machine. Additionally, an in vitro dissolution evaluation was performed in accordance with the ISO 10993-14 standard [31].

Our study adds important information regarding the proliferation of MG63 osteoblast-like cells at a longer time, after 48 h, 72 h, 96 h, and 168 h. Furthermore, we performed inverted microscopic images of MG63 osteoblast-like cells on various surfaces: non-treated plates, TC-treated plates, and BG. Our microscopic images revealed spheroid formation, detailing the surface-dependent behavior and survival patterns of these cells.

An important conclusion can be drawn: immediate adhesion is not a prerequisite for cell survival, as cells were able to form and proliferate in spheroids. Typically, adherent cell types such as MG63 undergo cell death within 24 h if they are unable to adhere [32].

The question arises as to whether the proliferation and the spheroid-forming property of osteoblast-like cells can be attributed to the effects of the bioglass. Based on the data of Figure 7 and the inverted microscopic images, this seems unlikely. The cells proliferate slightly slower on the bioglass surface compared to the positive control well, and a similar tendency is observed in the proliferation of MG63 cells cultured without bioglass granules.

It is likely that this phenomenon is facilitated by substances released from the bioglass. However, it is also possible that these osteosarcoma-derived cells have an inherent tendency to form spheroids spontaneously, enabling them to survive for days without an adherent surface. Data from the literature also support this hypothesis, as MG63 cells, derived from human osteosarcoma, have the capacity to form spheroids and survive under anchorage-independent conditions under appropriate culture conditions, a characteristic common among cancer cell lines. The appearance of spheroids generally indicates insufficient cell adhesion, and the use of non-adherent plates can further promote spheroid formation. In spheroid models, cells aggregate to form a three-dimensional (3D) structure, better mimicking the tumor microenvironment compared to traditional two-dimensional (2D) cultures. The advantages of spheroid models are that they better replicate the behavior of real tissues, including nutrient gradients and cell–cell interactions. MG-63 cells in spheroid form exhibit increased expression of bone-related proteins and vascular endothelial growth factor (VEGF). Consequently, we can conclude that hypoxic cores may develop in larger spheroids, similar to solid tumors [33,34,35,36].

## 5. Conclusions

Considering the results, it can be concluded that the bioglass tested in our study is suitable for implantation in bony environments, due to its favorable non-cytotoxic properties. Our findings are in accordance with previous works and show that bioglass is a good substrate (or environment) for MG63 cell survival and proliferation.

The main limitations of this investigation are its two-dimensional cell culture in vitro feature and use of an osteoblast-like cell line. To fully establish the biocompatibility and suitability of the tested bioglass as a potential orthopedic material, further in vitro and in vivo tests are needed. Therefore, in continuation of this study, we plan to perform a 3D cell culture experiment with primary human osteoblast and fibroblast cells. These can be continued with in vivo studies, to ultimately confirm their biocompatibility.

The clinical relevance of the study is multifaceted. On one hand, as discussed in the introduction, bioglass can be clinically useful for the replacement of bone defects, whether septic or aseptic. An important development could be the incorporation of antibacterial particles (Ag, Au, or Zn) into the BG pore structure, which will reduce the infection rate of these implants. On the other hand, bioglass has potential applications in implantology. For example, implants, including prostheses, could be coated with it. This silicate coating facilitates the formation of an apatite layer on the surface, enhancing biocompatibility. In addition, the bioglass coating increases bond strength, which supports the in vivo integration of the implant. This leads to reduced stress at the implant–tissue interface and improved implant stability and longevity.

## Figures and Tables

**Table 1:** Average OD values measured at 450 nm ± SE for MG63 osteoblast-like cells cultured on bioglass.

Time (h)	24 h	48 h	72 h	96 h	168 h
**OD ± SE**	0.254 ± 0.004	0.362 ± 0.014	0.546 ± 0.043	0.952 ± 0.094	1.518 ± 0.072

**Table 2:** Pairwise comparisons of groups (*p* values) by the Kruskal–Wallis test with SPSS 29 program (IBM, New York, NY, USA). The probability value of *p* < 0.05 was considered statistically significant. Asterisks denote the following significant differences: * *p* < 0.05, ** *p* < 0.01, and *** *p* < 0.001; N.S.: non-significant.

*p* Values, Pairwise Comparisons of Groups Kruskal–Wallis Test					
Sample 1 vs. Sample 2	24 h	48 h	72 h	96 h	168 h
MEM vs. MEM + BG	0.976 N.S.	0.943 N.S.	0.972 N.S.	0.972 N.S.	0.619 N.S.
MEM vs. MEM + BG + MG63	* 0.012	** 0.006	** 0.006	** 0.007	** 0.004
MEM vs. MEM + MG63	*** *p* < 0.001	*** *p* < 0.001	*** *p* < 0.001	*** *p* < 0.001	*** *p* < 0.001
MEM + BG vs. MEM + BG + MG63	* 0.014	* 0.013	* 0.012	* 0.012	* 0.015
MEM + BG vs. MEM + MG63	*** *p* < 0.001	*** *p* < 0.001	*** *p* < 0.001	*** *p* < 0.001	*** *p* < 0.001
MEM + BG + MG63 vs. MEM + MG63	* 0.014	* 0.035	* 0.034	* 0.034	* 0.034

## Data Availability

The original contributions presented in the study are included in the article, further inquiries can be directed to the corresponding author.
